# Ecosystem service evaluation based on local knowledge of residents using spatial text-mining

**DOI:** 10.1038/s41598-023-49612-1

**Published:** 2023-12-20

**Authors:** Jae-hyuck Lee, SoEun Ahn

**Affiliations:** Korea Environment Institute, Sejong, Republic of Korea

**Keywords:** Ecology, Environmental social sciences

## Abstract

This study aims to evaluate the ecosystem services of Upo wetland, one of the best-known Ramsar sites in Korea, reflecting the characteristics of the ecological assets and local knowledges in the area. Application of spatial text-mining begins with collecting local perceptions and knowledge of residents on the 17 ecological assets of Upo site and surrounding area. Our results identified five important ecosystem services: flood control during heavy rainfall, water purification by aquatic plants, cultural and natural heritages, agricultural products and water provision for crop cultivation. GIS created a map where these ecosystem services were linked to the locations of 17 ecological assets. This map showed which ecosystem service is associated with particular ecological assets and their characteristics from residents’ perspectives. Mapping local knowledge using the spatial text-mining is able to identify multi-functional bases which provide various ecosystem services in the same location simultaneously. Identification of multi-functional bases can provide information for local government to design an effective and comprehensive management plan considering physical-cultural geography of ecosystem services.

## Introduction

In the local level, ecosystem service evaluation involves confirming the characteristics of ecological assets and supporting environmental planning to enhance the provisions of ecosystem services in the area^1^. For this task, researchers have advocated for and attempted to evaluate ecosystem services through the local knowledge of the residents on the ecological characteristics of nature^2–4^. The residents have held the knowledge regarding ecological assets for a relatively long period, indicating that they have formed their own perceptions about the relationship between people and nature^5^. By revealing the characteristics of ecosystem functions and services in the particular region, local knowledge can play an important role in establishing conservation policy and ensuring sustainable provisions of ecosystem services and benefits people rely on.

Local knowledge, by nature, are likely to be summarized in qualitative ways and can complement the data-driven evaluation of ecosystem services such as indicator and modeling approaches^6^. Family of participatory methods can provide a useful framework to gather information by employing techniques such as personal or focus group interviews and stakeholder workshops. Evaluation of cultural ecosystem services has been a main domain for this line of research^7^.

However, despite the advantage of active stakeholder-engaging in the process, participatory methods with qualitative data are largely limited to represent the results from the evaluation of ecosystem service in space. Research on the local knowledge of ecosystem service have contributed significantly to validating primary ecosystem services, it is hard to understand geographical distribution of ecological assets and ecosystem services they provide due to the nature of qualitative data. However, from the perspective of environmental planning, ecological assets in which ecosystem services are produced must be understood within the surrounding natural environment; therefore, it is necessary to confirm the geographical context^8^.

The purpose of this study is to identify and evaluate the important ecosystem services of Upo wetland, one of the best-known Ramsar sites in Korea, based on the local perceptions and knowledge of residents. Upo wetland is located in Nakdong river basin of South Korea and designated as protected area in 1999. Spatial text-mining, where morphological analysis, factor analysis and GIS mapping are conducted in order, is adopted to examine the qualitative data. Spatial text-mining begins with collecting local perceptions and knowledge of residents on the ecological assets and ecosystem services of Upo site. The resident survey is conducted by the Upo Ecotourism Association. This study aims to perform spatial text-mining on local knowledge data^9^ to create an evaluation map of ecosystem service where ecological assets are linked to and tries to provide information for environmental planners to understand the ecosystem functions and services based on the ecological assets in the region. Accordingly, this study attempted to realistically evaluate the local characteristics by collecting residents' local knowledge and applying the spatial text mining technique to quantify and map the local knowledge for each ecosystem service category.

## Literature reviews

### Participatory ecosystem service evaluation

Residents are most affected by the environmental changes close to them and their local knowledge can be effective in finding a list of ecological assets. Through residents’ participation techniques, aspects such as climate change and biodiversity loss can be confirmed, data for environmental management can be produced, and residents' environmental capacity can be enhanced^10^. Furthermore, resident science can enable the democratization of environmental information^11^ and change a broad range of ideas of residents into prevailing social norms^12^. In particular, among the forms of resident participation, participatory geographical information systems (PGIS) can be evaluated as a practical framework for participation, and the results can be provided as a map^13^. PGIS has evolved rapidly with the development of map-based websites and mobile phones. As a result, more people can easily participate in data generation, and a foundation has been laid for researchers to collect relevant information efficiently. In addition, evaluating ecosystem services with resident participation could be established as a more effective and efficient method.

Ecosystem service evaluation with residents' participation effectively reveal crucial information in regions with unavailable environmental data. Moreover, it is less expensive than acquiring ecological data, has educational value for participating residents, and assists in identifying local ecosystem characteristics. It also provides crucial data for decision-making in stakeholder cooperation processes^14^. Using this method, researchers have evaluated the current state of significant indicators^15,16^, predicted the future state of projects, and compared them with the present state described by locals^17,18^. These evaluation promptly identified the local conditions, discovered multifunctional spaces^19^, and evaluated and mapped the distribution of ecosystem service functions^20^.

Through these evaluations, researchers have identified hotspot areas for the four ecosystem service categories (crop production provisioning services, air and water quality regulating services, animal and plant habitat supporting services, and leisure and recreation cultural services). By including resident participation in ecosystem service evaluations, Lee^21^ found that support services mainly occur in the mountains and rivers, cultural services in urban centers with large populations, and regulating services in the area between the foot of mountains to the streams. It presented environmental plans and management measures to revitalize these services. However, these approaches require additional qualitative explanations to identify the respective functions of the ecosystem types in the field. For example, additional information was necessary to explain which animals and plants exist as support services, the reasons for regulating services, and region-specific categories, such as spiritual values for cultural services^22^.

An alternative approach is investigating ecosystem services through in-depth interviews with residents. This technique involves seeking responses to semi-structured questions regarding ecosystem services and then analyzing the results. Researchers evaluated cultural services with this method and found various cultural services according to local characteristics. Nonetheless, as the analysis results were not quantified, the researchers’ subjective judgment obscured the findings^7^.

### Spatial text mining

Text mining is garnering increased attention as a technique to quantify verbal data. This method involves structuring key content based on the occurrence frequency of keywords. It helps visualize the key messages^23,24^. Therefore, text mining techniques are used in many social science areas to quantify qualitative data and display the results. Studies in the ecosystem services field have also investigated residents' knowledge of cultural service category characteristics according to assets in text and quantified them through text mining; however, they could not map the data or reveal the ecological context^22^.

Text mining produces quantified numbers, which can be used for mapping. This method has been defined as ‘spatial text mining’^9,25^. Through a factor analysis between spaces and significant words, this technique can identify the main keywords used in each response and the characteristics of each distribution. Spatial text mining has the advantage of being able to quantify and map participants’ knowledge of distribution. Hence, it is a highly effective technique for structuring and mapping residents’ knowledge. Researchers have attempted to evaluate cultural services using the text-mining technique. However, rather than representing residents’ in-depth local knowledge, they only analyzed their leisure behavior via hashtags attached to photos; therefore, the local ecosystem’s functions were not identified in depth^26^.

## Methodology

### Research site

**Figure 1:**
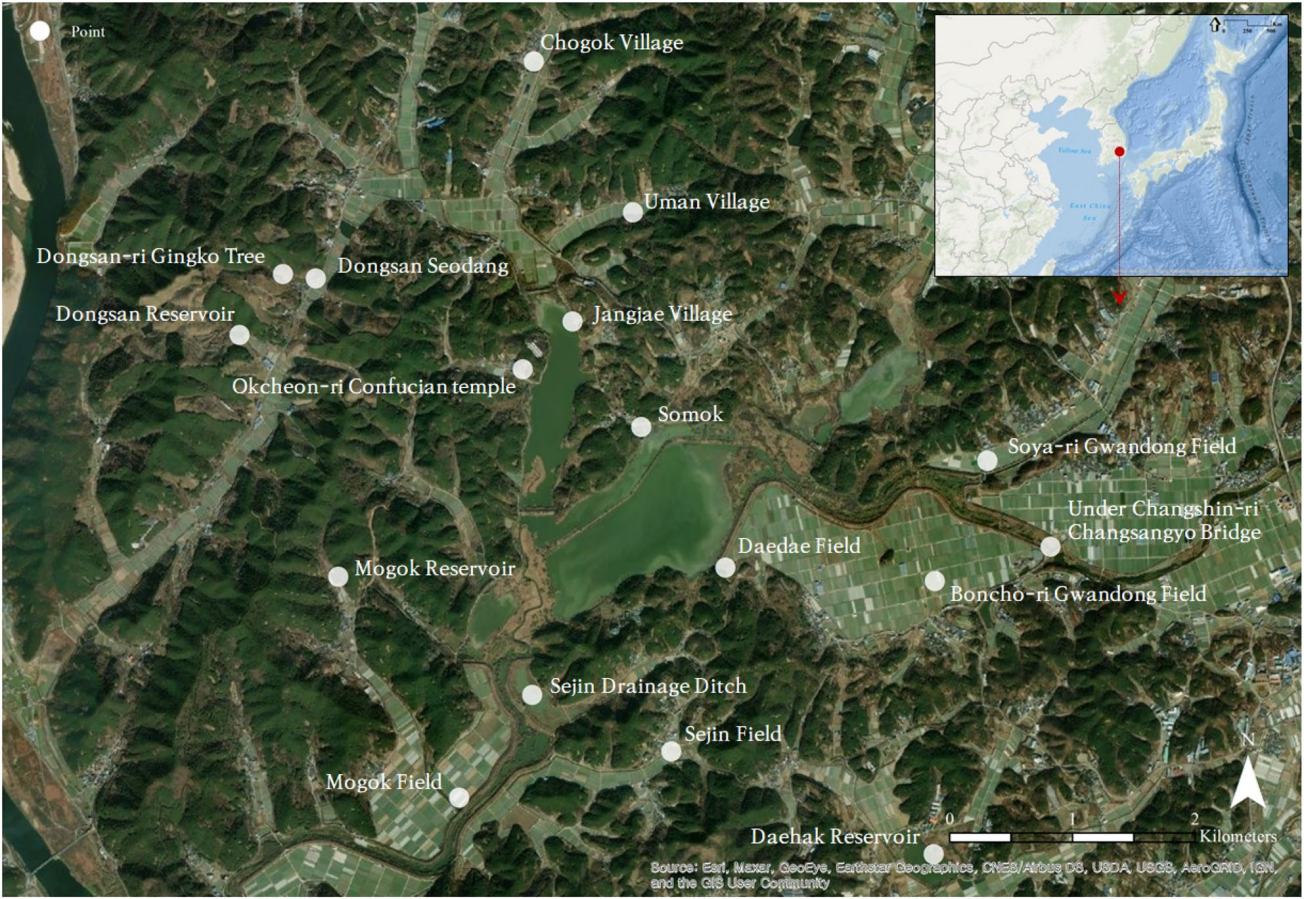
Ecological assets selected by the Upo Ecotourism Association. *Source* of background satellite map: Google Earth.

Upo Wetland (35°33′ N, 128°24′ E), located in Changnyeong County, South Korea, near the Nakdong River, was selected as the study site. As a representative wetland protected by the Ramsar Convention, Upo provides a habitat for various wild animals and plants. It is a conservation area with hydrological value; water is stored in the wetland soil during the rainy season or floods and then continuously supplied to the surrounding area during the dry season^27,28^. Based on these ecological assets, conflicts occur in the Upo Wetland because stakeholders value ecosystem services differently depending on their interests. For instance, the environmental groups want to preserve the ecosystem as it is, the local governments promote eco-tourism to increase revenues in the region, farmers prefer to earn income from paddy farming and fishermen wish to profit from fishing activities. In particular, a levee in the south of Upo regulates the water level of Upo. Fishermen want this levee for a higher water level to float their boats and efficient fishing. However, environmental organizations oppose it and want the fish in the Upo wetland to move freely. In addition, during floods, the water overflows the dike and floods the farming area. Farmers are demanding the construction of a cement dike to prevent this, but environmental organizations and local governments oppose it because of ecological conservation. The provincial government plans to generate a lot of revenue by promoting ecotourism. But, residents are concerned about environmental degradation and damage to fisheries and agriculture caused by tourists. As such, there are many conflicts in Upo due to different ecosystem service needs, and solutions are needed. Therefore, this study aimed to collect and map ecological knowledge corresponding to ecosystem services in Upo, identify spatially characterized ecosystem services, and propose a plan to manage stakeholders' needs through harmonious spatial planning. For this purpose, seventeen primary ecological assets were identified through the Upo Ecotourism Association, a representative regional environmental group, for collecting ecological perception (See Fig. 1).

### Research methods

**Figure 2:**
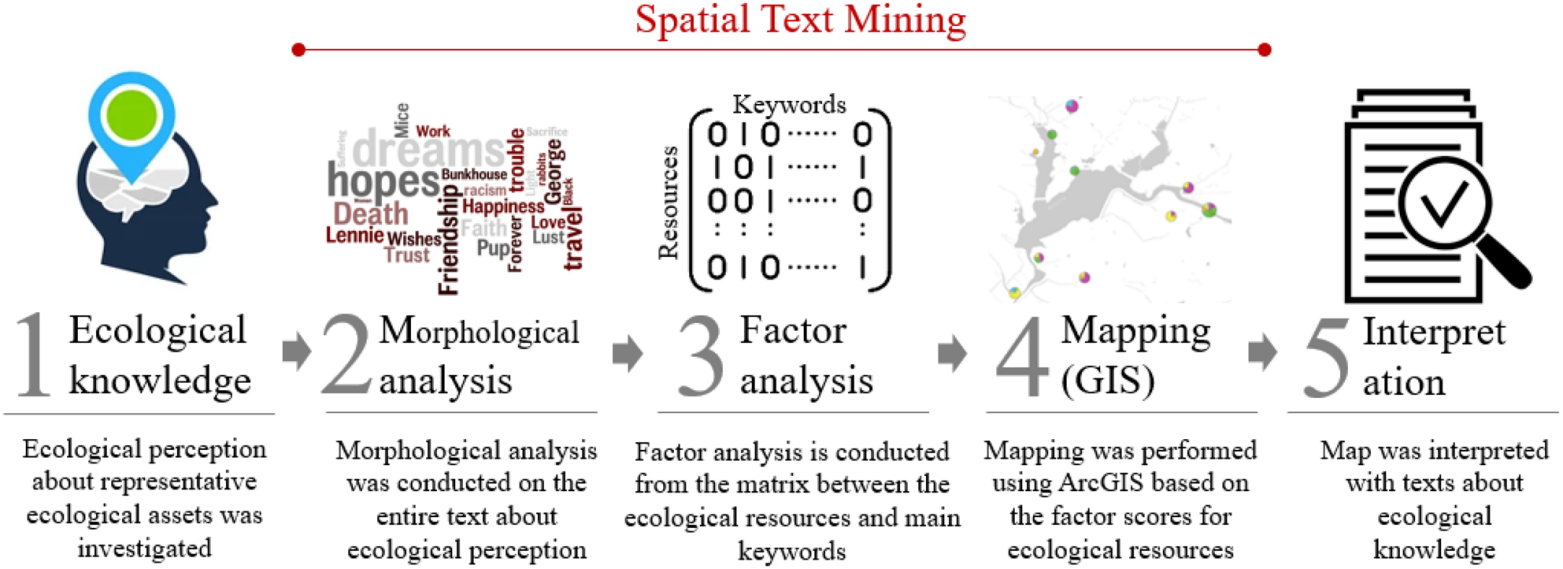
The research steps are undertaken in this study.

This study used spatial text mining to spatially represent the local knowledge according to the respective ecosystem service. The technique was utilized to analyze and map ecosystem services expressed as local knowledge for each ecological asset in spatial text mining process based on factor analysis^9,29^. This study collected ecological knowledge, used spatial text mining in steps 2 through 4, and validated it in step 5. The detailed steps are shown in Fig. 2.

According to the ecosystem services framework, local knowledge about representative ecological assets was first investigated in collaboration with the ten staff of the Upo Wetland Ecotourism Association (http://www.upoecotour.net). This study used explanation data to rapidly evaluate ecosystem services created by Upo Ecotourism Association in 2019. The Upo Wetland Ecotourism Association was founded in 2014 by the residents of Upo Wetland and operates an ecotourism business to preserve the natural environment of the wetland, share the traditions and life of the swamp with visitors, and conserve and wisely use the natural ecology and landscape of Upo wetland. The participants first received a lecture on the basic concepts and indicators of ecosystem services and then were asked to select the primary ecological assets of Upo wetland. The selection criterion was that the primary ecological assets introduced to outsiders could provide sufficient ecological knowledge. Then, they were asked to write ecological knowledge for each ecological help by ecosystem service. In this process, they were asked to write the ecological use status of the area, not their opinions. The study was conducted by utilizing the explained reasons as the local knowledge of the ecological assets of residents. The study was conducted by utilizing the explained reasons as the local knowledge of the ecological assets of residents.

Secondly, morphological analysis NetMiner 4.3, a Korean text mining program, which breaks down a message into its most diminutive word form that has meaning, was conducted on the entire text about local knowledge in the ecosystem services framework. Frequently derived keywords were regarded as essential words, and the top 10% of the most frequent nouns, the words that can stand alone and have meaning, were selected as the primary keywords^30^ (See Table 1).

**Table 1:** Selected keyword.

Division	Keywords
Water related	Clean water(정수), Embankment(제방), Flood(홍수), Flooding(침수), Flow(흐름), Flow-rate(유속), Freshwater(담수), Inflow(유입), Inundation(범람), Outflow(유출), Reservoir(저수지), River(하천), Sedimentation(퇴적), Supply(공급), Surface water(지표수), Swamp(늪), Typhoon(태풍), Underground-water(지하수), Water(물), Water-quality(수질), Wet(습윤), Wetland(습지)
Biology related	Aquatic plants(수생식물), Ecology(생태), Food(먹이), Ginkgo tree(은행나무), Habitat(서식), Migratory bird(철새), Nature(자연), Reed(갈대), Silver grass(억새), Soil(토사), Tree age(수령)
Agriculture related	Agricultural water(농업용수), Cultivation(재배), Drainage(배수장), Farmland(농경지), Garlic(마늘), Onion(양파), Paddy field(무논), Rice paddy(논), Rice plant(벼)
Village related	Arable land(경작지), Chapel(제실), Shrine(사당), Single-clan(집성촌), Village(마을)
Miscellaneous	Control(조절), Effect(영향), Harm(피해), Improvement(개선), Prevention(예방), Winterization (월동), Wood(목본)

Selected Korean words may be redundant in the English translation.

A matrix was created between the ecological assets and the main keywords for factor analysis. This word frequency matrix between assets and keywords is presented in Appendix 1. Factor analysis can determine the keywords mainly used for each ecological asset. In addition, it performs standardization, thus identifying phenomena in which words frequently occur within a large quantity of text. This study used factor analysis due to these advantages^31^. The result shows the relationships between assets and keywords, which means the specialized function of the assets. Fourthly, the mapping was performed using ArcGIS 10.3 based on the factor scores for ecological assets. Lastly, based on the expressed map, the local knowledge-based ecosystem service evaluation map was interpreted, and ecosystem service functions were identified according to the characteristics of the local ecosystem. It presents the geographical contexts of the ecological assets’ ecosystem functions.

## Results from factor analysis and mapping

Five important ecosystem services were extracted from the factor analysis: flood control during heavy rainfall (factor 1: regulating service), water purification by aquatic plants (factor 2: regulating service), cultural and natural heritages (factor3: cultural service), agricultural products and water storage for crop cultivation (factor 4 & 5: provisioning services) (See Table 2). These ecosystem services are combined with local traditions and the Upo wetland has become a unique asset in the region.

**Table 2:** Total variance in ecosystem services.

Components	Initial eigenvalue			Extraction sums of squared loadings			Rotation sums of squared loadings		
	Total	% of variance	Cumulative rate (%)	Total	% of variance	Cumulative rate (%)	Total	% of variance	Cumulative rate (%)
1 Flood control	4.43	26.05	26.05	4.43	26.05	26.05	3.48	20.49	20.49
2 Water purification of aquatic plants	3.07	18.03	44.08	3.07	18.03	44.08	3.11	18.30	38.79
3 Heritage	1.85	10.85	54.93	1.85	10.85	54.93	2.04	12.02	50.81
4 Agriculture	1.49	8.74	63.67	1.49	8.74	63.67	1.89	11.10	61.91
5 Water storage	1.25	7.36	71.04	1.25	7.36	71.04	1.55	9.13	71.04

Extraction method: Principal component analysis.

Table 3 presents the key words and associated ecological-assets by each factor with the loading scores. Characteristics of arable land is crucial to understand the flood control. The arable lands around the villages, which hold the water during heavy rainfall, can protect the villages from the flood. The wet arable lands with cultivating crops improve water quality and help soil sedimentation as well (factor 1). Wetland provides suitable conditions for various aquatic plants to grow because the water flows are slowed down. These plants treat the nutrients in the water and enhance the water quality. In addition, vegetations in the water provide habitats for fish and birds (factor 2). The ancestral shrine and 500-year-old Gingko tree near the Dongsan Seodang are representative cultural and natural heritages identified in the area (factor 3). Agricultural products such as rice, garlic and onions are regarded as important provisioning service in the area and the water supply to cultivate these crops seems to be important as well (factor 4 and 5).

**Table 3:** Primary keywords and target site distribution according to different factors.

Factor	Keyword	Loading	Target site	Loading	Service description
1 Flood control	Village	4.565	Uman Village	0.903	Uman Village’s rationale was as follows: “Wet arable land performs freshwater function during intensive rainfall, regulating flooding; crops grown in wet arable land function to purify water and improve water quality, and soil sedimentation also occurs.”
	Wet	2.851	Soya-ri Gwandong Field	0.856	
	Arable land	2.839	Chogok Village	0.845	
	Freshwater	1.318	Sejin Field	0.817	
			Sejin Drainage Ditch	0.522	
2 Water purification of aquatic plants	Water	5.170	Jangjae Village	0.919	Somok rationale was as follows: “Aquatic plants in wetlands influence water purification and water quality improvement, and sedimentation occurs owing to slow flow rate.”
	Wetland	2.334	Somok	0.919	
	Aquatic plants	1.671	Changsan Bridge	0.768	
	Swamp	1.539	Mogok Reservoir	0.606	
	Habitat	1.292			
3 Heritage	Single-clan village	5.175	Dongsan-ri Ginkgo Tree	0.818	Dongsan-ri Ginkgo Tree’s rationale was as follows: “a 500-year-old tree designated as a protected tree; a living fossil.”
	Tree age	3.089	Dongsan Seodang	0.725	
	Shrine	2.147	Okcheon-ri Confucian Temple	0.580	
	Chapel	1.391			
	Village	1.369			
4 Agriculture	Rice paddy	2.610	Mogok field	0.805	Mogok rationale was as follows: “Mogok field was formed after Mogok embankment was built: rice, garlic, and onion cultivation.”
	Rice plant	2.353	Boncho-ri Gwandong Field	0.720	
	Embankment	1.981	Daehak Reservoir	0.517	
	Onion	1.822			
	Garlic	1.817			
	Water	1.609			
	Farmland	1.416			
	Food	1.341			
	Cultivation	1.079			
	Village	1.037			
5 Water storage	Agricultural water	3.598	Dongsan Reservoir	0.834	Dongsan Reservoir’s rationale was as follows: “maintains water quantity and supplies agricultural water together with nearby reservoirs.”
	Soil	2.474	Mogok Reservoir	0.502	
	Reservoir	2.109			
	Water	1.853			
	Sedimentation	1.775			
	Supply	1.301			
	Cultivation	1.341			

**Figure 3:**
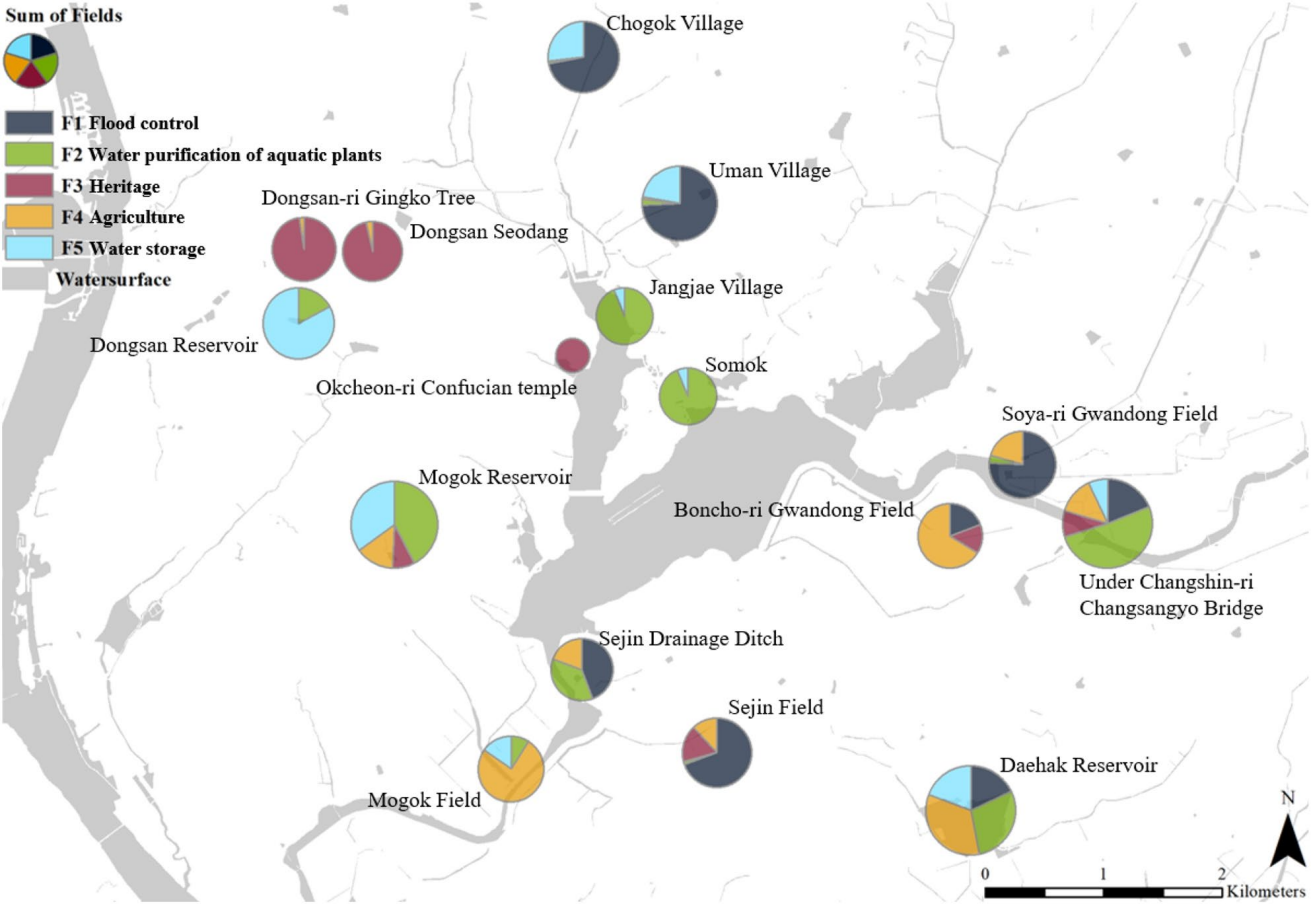
The maps linking ecosystem services and the locations of ecological assets in the Upo area.

To investigate the contexts of physical-cultural(or human) geography, the map was created where the five ecosystem services identified and the sixteen locations of ecological assets are linked. The phi-chart legend in the Fig. 3 shows the sum of loading scores and proportions of five ecosystem services for the corresponding ecological assets. Water purification and habitat support are mainly located around Upo in the middle indicating that these services are largely depended on the Upo itself. Flood control is connected to the Chogok and Uman villages near the streams in the northern part of Upo. Agricultural products are linked to the fields such as Boncho-ri Gwandong and Mogok filed which are located in the southern part of Upo. Cultural and natural heritages are connected to the Dongsan Seodang and Dongsan-ri village which are located in the north-western part of Upo.

The results are understood that, from the physical-geography perspectives, the foothills and tributaries with vegetations are apt to prohibit the flood, the Upo waterbody itself performs water purification through aquatic plants, the traditional villages combined with ecological asset create cultural services and rich rice fields are formed by Upo wetland over the years and produce agricultural products.

If the goal of local government is to strengthen single individual ecosystem service, information summarized in the Fig. 3 can provide useful information in terms of where and what needed to be focused on. For instance, cultural services can be enhanced in the villages west of Upo by introducing various eco-tourism and environmental education programs. Agricultural productions including rice can be increased by constructing a small bank which controls both the quantity and quality of water for irrigation in the southeast. Flood control in the upstream of Upo can be targeted by maintaining the functions of wet arable land. Water purification and habitat provision can be improved in the center of Upo by managing aquatic vegetations to support these functions.

## Discussion: environmental planning for multi-functional bases with spatial text-mining

In addition to providing information for local government to improve single individual ecosystem services, mapping local knowledge using the spatial text-mining is able to identify multi-functional bases which provide various ecosystem services in the same location simultaneously^19^. Contrast to environmental planning which focuses on improving particular single ecosystem service, identification of multi-functional bases can provide information for local government to design an effective and comprehensive management plan considering physical-cultural geography of ecosystem services involved in the area.

Our results demonstrate that the Upo has created wet arable lands over the years and provided various water-related regulating ecosystem services such as flood control, water purification and habitats for fish and birds. Local traditions coupled with the nature have created unique cultural heritages and landscapes. As a result, Upo wetland becomes one of the best-known sites for both conservation and eco-tourism in Korea indicating that a balanced management plan is required.

**Figure 4:**
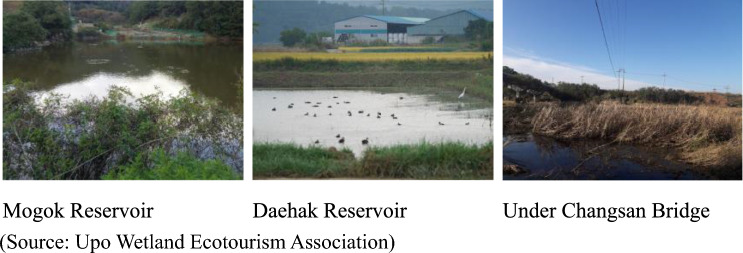
Photographs of the multifunctional bases.

To illustrate how our case study can be used for this purpose, we identified three multi-functional bases: Mogok Reservoir, Daehak Reservoir and Changsan Bridge in Fig. 4 where five ecosystem services are relatively evenly distributed. The main services Mogok Reservoir provide are controlling water quality and storing the water for agriculture. The rice fields near the Daehak Reservoir are known to provide a shelter for migratory birds. Under the Changsan Bridge, various aquatic plants such as reeds and mugwort improve water quality. Fish and birds that feed on aquatic plants live there. Water is used for agriculture as well.

Significant multifunctional ecological assets are located in water-related points.

Further understanding the characteristics of these multi-functional bases helps to design a plan to manage ecological assets and ecosystem services. Moreover, multi-functional bases can serve as centers for management of various ecosystem services they provide simultaneously. Managing multi-functional bases require the plan where the supply of provisioning, regulating, cultural and supporting services are well balanced. Our case study shows that local perceptions and knowledge of residents obtained by spatial text-mining with mapping can guide the environmental planning where the provision of ecosystem services and conservation of ecological assets are managed in sustainable manners.

The method presented in this study is able to map ecological knowledge. It provides valuable information to understand the physical-cultural(or human) geography in the form of ecological knowledge^30^. Contrast to supply-driven evaluations such as modeling and indicator approaches, the knowledge-based evaluations are suitable to recognize, from user’s perspectives, that how ecosystem services are produced and what ecological assets are lined to. This information, thus, enables decision makers to identify ecosystem functions as well and can lead to design policies that enhance ecosystem services^32^ using the perceptions and knowledge of real users in the region^33^.

Another advantage of adopting the knowledge-based evaluation with mapping is to assist environmental planners to differentiate strategies where particular ecosystem services is matched with particular ecological asset^34^. Beyond improving ecosystem services, these methods can be utilized to develop the comprehensive spatial-planning in the region where environmental, social and economic objectives are well balanced. Considering that the sustainability can be an ultimate goal, the knowledge-based evaluations are effective tools to recognize and organize local knowledge on ecological assets and ecosystem services and are apt to transfer corresponding knowledge to the next generation.

In addition, mapping ecological knowledge can be an educational process to increase the awareness of local conservation initiatives^35^. For example, the residents who participate in the practice are more likely to better understand the area by mapping their ecological knowledge and other residents and tourists are able to recognize the local ecosystem by looking at the map as well^21^. The raised awareness about the ecological assets itself, including both residents’ and visitors’, is valuable resource to develop a model such as Education for Sustainable Development (ESD).

Note that a limitation exists in which only a part of residents participated in this study and, therefore, a caution is needed not to extend our results to any universal conclusion. The participation of more and diverse stakeholder groups, including the managers of local government, environmental researchers, environmental NGOs and visitors, will clearly improve the extend of knowledges from various perspectives on the local ecosystem resulting a more comprehensive and inclusive management plan. Participatory methods with mapping including spatial text-mining have great potentials in terms of involving various stakeholders in the valuation process, understanding the perceptions and knowledges on the relationship between nature and human, locating local ecological assets and ecosystem services. The research is warranted to develop more systematic procedure^36^ and proper tools to verify the qualitative data obtained^37^. Lastly, there is a certain possibility of linking participatory methods with mapping to other evaluation methods. One example is DMV(deliberative monetary valuation) where participatory methods are combined with monetary valuation method^38,39^.

## Conclusions

This study is significant because we use spatial text mining with resident participation as a method of ecosystem service assessment to map local knowledge regarding ecosystem services. Compared to the existing methods of ecosystem service assessments with resident participation, our approach identified the specific functions and distribution of ecosystems using critical words of the local sites. This effectively revealed the local site characteristics, which will provide essential data for environmental planning. Nevertheless, as the results of this study were obtained only from residents’ opinions, further verification by scientists is required. Future studies will include developing a proposed methodology that can systematically verify ecosystem service assessments with resident participation by scientists.

## Appendix

**Table:** Appendix 1. Word frequency matrix between assets and keywords.

Division	Keyword	Daehak Reservoir	Sejin Drainage Ditch	Sejin Field	Daedae Field	Jangjae Village	Uman Village	Chogok Village	Somok	Boncho-ri Gwandong Field	Changsan Bridge	Soya-ri Gwandong Field	Mogok Reservoir	Mogok field	Dongsan-ri Ginkgo Tree	Dongsan Reservoir	Okcheon-ri Confucian Temple	Dongsan Seodang
Water-related	Clean water	1	0	0	1	1	1	1	1	0	0	1	0	0	0	0	0	0
	Embankment	0	2	0	1	0	0	0	0	1	1	0	0	2	0	0	0	0
	Flood	0	1	1	1	0	1	1	0	0	1	1	0	0	0	0	0	0
	Flooding	0	0	0	1	0	1	1	0	2	0	0	0	0	0	0	0	0
	Flow	0	1	1	1	0	0	0	0	0	0	1	0	0	0	0	0	0
	Flow rate	0	0	0	0	2	0	0	2	0	0	0	0	0	0	0	0	0
	Freshwater	0	1	1	1	3	2	2	3	0	0	1	0	0	0	0	0	0
	Inflow	0	3	0	1	1	0	0	1	0	1	1	0	0	0	0	0	0
	Inundation	1	1	0	0	1	0	0	1	0	0	0	0	0	0	0	0	0
	Outflow	0	1	0	1	1	0	0	1	0	0	1	0	0	0	0	0	0
	Reservoir	3	0	0	0	0	0	0	0	0	0	0	3	0	0	1	0	0
	River	0	2	0	1	0	0	0	0	0	0	1	1	0	0	0	0	0
	Sedimentation	0	0	0	0	1	1	1	1	0	0	0	0	0	0	2	0	0
	Supply	2	0	0	1	0	0	0	0	0	0	0	0	1	0	2	0	0
	Surface water	0	1	1	1	0	0	0	0	0	0	1	0	0	0	0	0	0
	Swamp	0	2	1	4	4	0	0	4	0	1	1	0	1	0	0	0	0
	Typhoon	0	0	0	1	0	1	1	0	1	0	0	0	0	0	0	0	0
	Underground water	0	0	0	0	1	1	1	1	0	0	0	0	1	0	0	0	0
	Water	4	4	0	2	9	2	2	9	0	4	1	4	2	0	2	0	0
	Water quality	1	0	0	1	1	1	1	1	0	0	1	0	0	0	0	0	0
	Wet	0	2	2	2	0	3	3	0	0	0	2	0	0	0	0	0	0
	Wetland	1	0	0	0	6	0	0	6	0	1	0	1	0	0	0	0	0
Biology related	Aquatic plants	0	0	0	0	5	0	0	5	0	1	0	0	0	0	0	0	0
	Ecology	1	0	0	0	2	0	0	2	0	0	0	0	0	0	0	0	0
	Food	0	0	0	2	0	0	0	0	1	0	0	0	1	0	0	0	0
	Ginkgo tree	0	0	0	0	0	0	0	0	0	0	1	0	0	0	0	2	0
	Habitat	0	1	1	0	3	0	0	3	0	2	0	0	0	0	1	0	0
	Migratory bird	1	0	0	0	2	0	0	2	1	0	0	0	1	0	0	0	0
	Nature	0	0	0	0	1	0	0	1	0	0	0	2	0	0	0	0	0
	Reed	0	0	0	0	1	0	0	1	0	1	0	0	0	0	0	0	0
	Silver grass	0	0	0	0	1	0	0	1	0	1	0	0	0	0	0	0	0
	Soil	0	0	0	0	1	1	1	1	0	0	0	0	0	0	3	0	0
	Tree age	0	0	0	0	0	0	0	0	0	0	0	0	0	1	0	2	0
Agriculture related	Agricultural water	0	0	0	0	2	1	1	2	0	2	0	2	0	0	4	0	0
	Cultivation	1	0	0	1	0	1	1	0	0	0	1	0	2	0	1	0	0
	Drainage	0	3	0	0	0	0	0	0	0	0	0	0	0	0	0	0	0
	Farmland	1	1	0	1	1	1	1	1	1	2	1	2	1	0	0	0	0
	Garlic	1	1	1	1	0	1	1	0	1	0	1	0	2	0	1	0	0
	Onion	1	1	1	1	0	1	1	0	1	0	1	0	2	0	0	0	0
	Paddy field	0	0	0	3	0	0	0	0	0	0	0	0	0	0	0	0	0
	Rice paddy	1	1	1	3	0	0	0	0	2	0	1	0	1	0	0	0	0
	Rice plant	2	1	1	2	0	1	1	0	1	0	2	0	2	0	0	0	0
Village related	Arable land	0	2	2	3	0	3	3	0	0	0	2	0	0	0	0	0	0
	Chapel	0	0	2	0	0	0	0	0	0	0	0	0	0	0	0	0	1
	Shrine	0	0	0	0	0	0	0	0	0	0	0	0	0	0	0	3	0
	Single-clan	0	0	1	0	0	0	0	0	1	1	0	1	0	1	0	1	1
	Village	3	3	6	0	1	3	2	1	2	3	5	0	0	0	0	0	0
Miscellaneous	Control	0	1	0	0	1	1	1	1	0	0	0	0	0	0	0	0	0
	Effect	1	2	1	1	2	1	1	2	0	0	1	0	0	0	0	0	0
	Harm	0	0	0	1	0	0	0	0	1	1	0	0	0	0	0	0	0
	Improvement	1	0	0	1	1	1	1	1	0	0	1	0	0	0	0	0	0
	Prevention	0	1	1	1	0	0	0	0	0	0	1	0	0	0	0	0	0
	Winterization	0	0	0	0	2	0	0	2	0	0	0	0	0	0	0	0	0
	Wood	0	0	0	0	2	0	0	2	0	0	0	0	0	0	0	0	0

## Data Availability

The datasets generated during the current study are not publicly available due to data protection agreements (interviews) and the ongoing embargo period (survey data) but are available from the corresponding author upon reasonable request.
